# A Method for the Direct Identification of Differentiating Muscle Cells by a Fluorescent Mitochondrial Dye

**DOI:** 10.1371/journal.pone.0028628

**Published:** 2011-12-09

**Authors:** Tetsuaki Miyake, John C. McDermott, Anthony O. Gramolini

**Affiliations:** 1 Department of Physiology, University of Toronto, Best Institute Medical Research, Toronto, Canada; 2 Department of Biology, York University, Toronto, Canada; University of Udine, Italy

## Abstract

Identification of differentiating muscle cells generally requires fixation, antibodies directed against muscle specific proteins, and lengthy staining processes or, alternatively, transfection of muscle specific reporter genes driving GFP expression. In this study, we examined the possibility of using the robust mitochondrial network seen in maturing muscle cells as a marker of cellular differentiation. The mitochondrial fluorescent tracking dye, MitoTracker, which is a cell-permeable, low toxicity, fluorescent dye, allowed us to distinguish and track living differentiating muscle cells visually by epi-fluorescence microscopy. MitoTracker staining provides a robust and simple detection strategy for living differentiating cells in culture without the need for fixation or biochemical processing.

## Introduction

Much of our extensive knowledge of the molecular and cell biology of skeletal muscle differentiation is due to the availability of immortalized cell lines such as the C2 cell line, with its sub-variants including the C2C12 line. C2 cells were originally established from adult satellite cells [1], [2]. These cells can proliferate with high mitogenic stimuli and form multi-nucleated myotubes (MTs) readily upon reduction of mitogens. However, the differentiation process is not fully synchronized, and due to stochastic reasons, a significant portion of the population does not form differentiated MTs, remaining in a quiescent mono-nucleated state [3]. Therefore, the ability to separate these populations would be a great advantage in characterizing the molecular events during muscle differentiation. To identify terminally differentiating muscle cells, detection of muscle specific proteins by immuno-fluorescence (IF), immuno-chemistry or introduction of muscle specific gene promoter-reporter constructs are commonly used. However, fixation of the cells or transfection methods may limit downstream applications.

Muscle cells have highly specialized features including a robust mitochondrial network [4]. Here we report a useful method to identify differentiating muscle cells without disrupting the differentiation program. Staining mitochondria with a low toxicity cell permeable fluorescent dye and visualization with fluorescence microscopy allows detection of differentiating cells. Using this live-cell imaging modality, we were able to detect differentiating muscle cells with minimal invasive manipulation.

## Results

### Live cell mitochondrial staining exhibits high mitochondrial reactivity in myotubes but not undifferentiated cells

Since differentiated muscle cells contain an extensive mitochondrial network to support the energy demands of this tissue [5], [6], we hypothesized that detection of active mitochondria might allow us to distinguish differentiating muscle cells from non-differentiating muscle cells. In order to detect living muscle cells visually, we used a cell-permeable low toxicity fluorescent dye, MitoTracker Red CMX-Ros (Invitrogen), which stains mitochondria specifically and responds to changes in mitochondrial membrane potential [7]. Mitochondria in proliferating C2C12 cells in growth medium (GM; 10%FBS supplemented DMEM) were labelled with MitoTracker Red (50 nM) for 30 min at 37°C. To visualize the cell nuclei, we used cell-permeable and fluorescent DNA dye, bisBenzimide H 33342 trihydrochloride (1 µM Hoechst 33342; Sigma).

**Figure 1:**
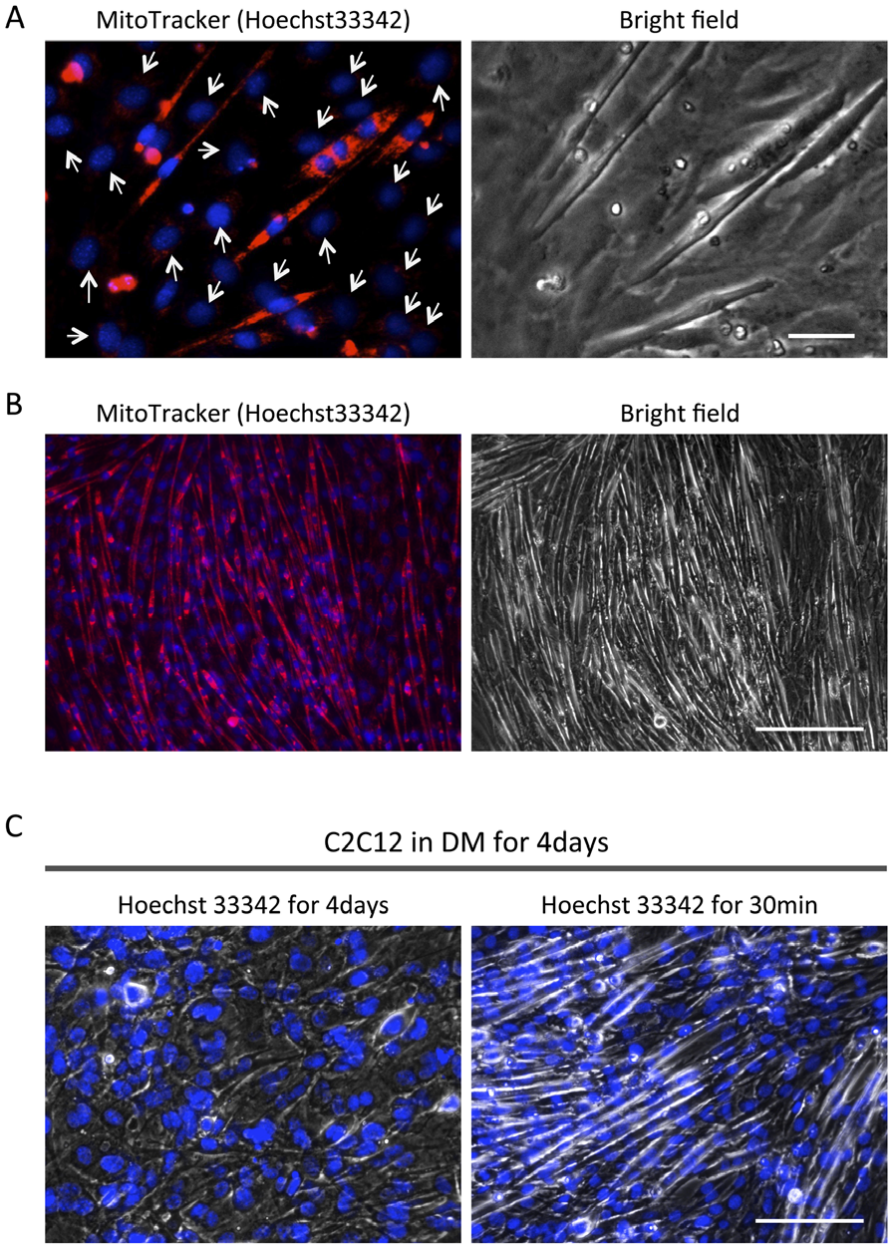
Live cell mitochondrial staining exhibits high mitochondrial reactivity in myotubes but not undifferentiated cells. C2C12 cells were seeded on the cell culture dishes. Upon reaching confluence, the cells were transferred to DM to induce MT formation. The DM was changed every 2 days. MitoTracker and Hoechst were added into the media for fluorescence staining of mitochondria and nuclei. After 30 min of incubation at 37°C, live-cell images were recorded by bright field or fluorescence microscopy at day2 (A) and day4 (B). The cells were kept for 4days in DM with/without Hoechst 33342 (1 µM), and then Hoechst was added to stain nuclei (C). Cell morphology was also recorded by bright-field phase-contrast microscopy. MitoTracker and Hoechst signals are shown in red and blue, respectively (scale bar in A = 10 µm, B = 100 µm, and C = 50 µm).

In order to test mitochondrial reactivity in differentiating cells to the MitoTracker, C2C12 cells were induced to differentiate in differentiation medium (DM; 2% FBS containing DMEM) for 4 days. Multi-nucleated MTs formed and some mono-nucleated cells were observed on the plate (data not shown). Double staining of mitochondria and nuclei was performed and all nuclei were visualized by Hoechst 33342 staining. In contrast, the mitochondria in the multi-nucleated MTs but not mono-nucleated cells were highly reactive with MitoTracker Red. As seen in Figure 1 (higher magnification in A (day2 in DM) and lower magnification in B (day4 in DM)), the nuclei (blue) of the undifferentiated cells (indicated by white arrow) are not surrounded by a signal from mitochondria (red). Since the differences in the red fluorescence signal intensities are large enough, in short exposure times, the signals from mitochondria in undifferentiated cells were much lower relative to that of MTs (Figure 1A, 1B). At day 2, some of the mono-nucleated cells were MitoTracker positive, but they show the typical morphological change in the differentiating cells, such as elongation (bright field micrographs, Fig. 1). In these experiments, however, we noted that addition of the Hoechst 33342 into the cell-culture medium resulted in inhibition of MT formation in longer treatments (Figure 1C).

### Differentiating cells are distinguishable by mitochondrial reactivity

**Figure 2:**
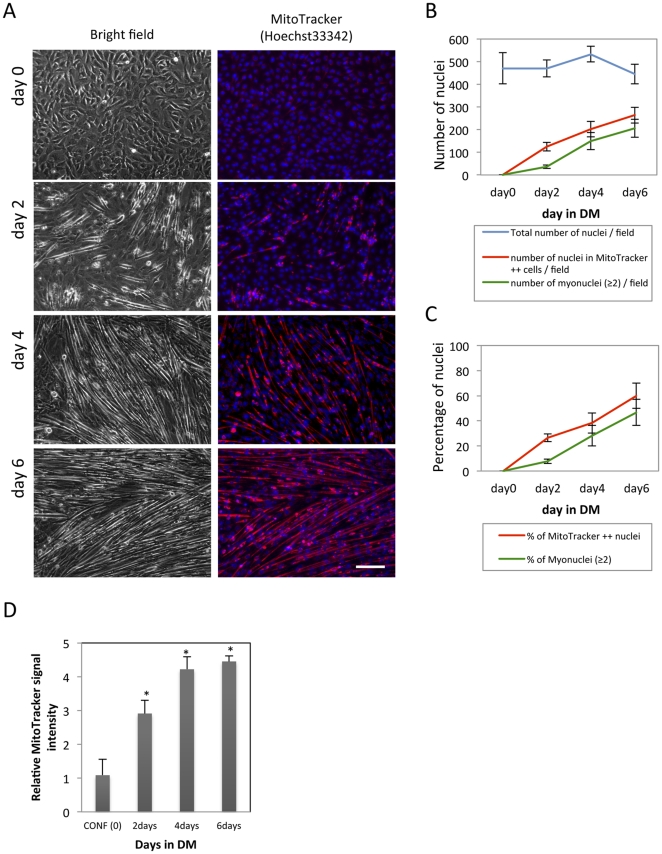
Differentiating cells are distinguishable by MitoTracker reactivity. C2C12 cells were seeded on the cell culture dishes and induced MT formation as described above. At the indicated day, the cells were double stained by MitoTracker and Hoechst 33342 as described above. Live-cell images were recorded by both bright field phase-contrast and fluorescence microscopy. MitoTracker and Hoechst signals are shown in red and blue respectively (A) (scale bar = 50 µm). Total number of nuclei, number of nuclei in the multi-nucleated (≥2) cells, and in MitoTracker positive cells per each micrograph were counted (n = 6). The average of each categories was graphed (mean ± st.dev) in panel B. (C) The percentage of nuclei in multinucleated (≥2) cells and MitoTracker positive cells over the total number of nuclei per field (n = 6) were calculated and graphed in C (mean ± st.dev) * (P<0.05 from confluent cells). (D) The same numbers of C2C12 cells were seeded on the cell culture dish (94-well plate (Nalge Nunc International)), and differentiation induced accordingly. Red fluorescent signal was measured by a plate reader, and the average values (n = 4) relative to that of the confluent condition (CONF (0)) were graphed (mean ± st.dev).

Next, we sought to determine when this mitochondrial reactivity change occurs during muscle differentiation. We double-stained nuclei and mitochondria as described above every two days following a culture media switch to DM and recorded MT formation by bright field phase-contrast and fluorescence microscopy. As seen in Figure 2A, as early as day2, even some of the mono-nucleated cells showed high MitoTracker reactivity (MitoTracker Positive Cells; MTP), and the population of MTP increased as MT formation progressing. Most MTPs, but not all, are multinucleated MTs in later time points. MTPs in early time points, for example at day2, are often not multi-nucleated. However, these cells clearly showed signs of early differentiation such as cell elongation compared to the flat, “cobble stone” morphology of the mono-nucleated cells as seen before (Figure 2A, day2). We quantified the degree of differentiation by counting the number of nuclei in multinucleated (≥2) cells and total number of nuclei in the field (n = 6). The total number of nuclei did not change significantly (Figure 2B) indicating the cells were not proliferating under these conditions. In agreement with morphological changes seen in the bright field micrographs, the number of nuclei in multi-nucleated cells and the percentage of nuclei in multi-nucleated cells over the total number of nuclei increased as differentiation progressed (Figure 2B and 2C). Here, both values change in the total number, and the percentage of the MTPs, show similar values to those of multi-nucleated cells (Figure 2B and 2C). As mentioned above, some of the MTPs were mono-nucleated. Therefore, the number of and the percentage of the MTPs were higher than those of multi-nucleated cells. We further quantified red fluorescent signal from MitoTracker during the course of muscle differentiation by a fluorescent plate reader. In agreement with our microscopy observations, the fluorescent signal intensity increased as the progression of muscle differentiation (Figure 2D). Therefore, MTP appears highly correlated with differentiating morphology and is easily quantifiable.

### MyHC and MyoG positive cells are also MitoTracker positive

**Figure 3:**
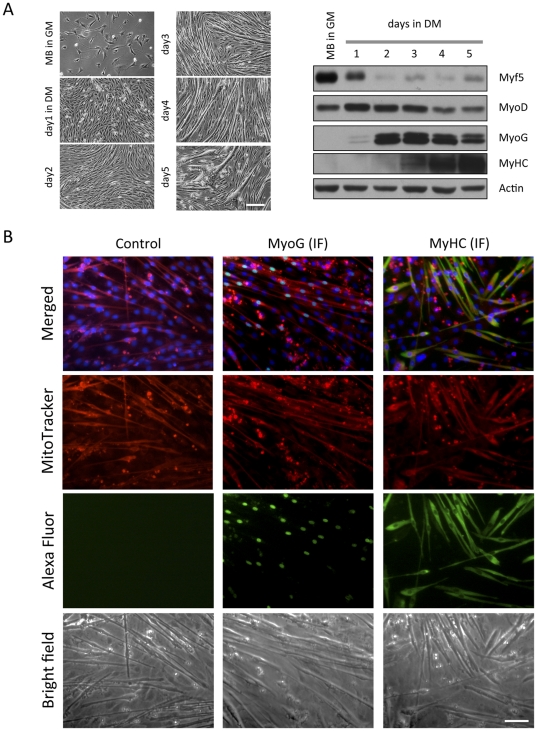
MyHC and MyoG positive cells are also MitoTracker positive. C2C12 cells were seeded on the cell culture dishes, and at the indicated day, live-cell morphology was recorded by a bright field phase-contrast microscopy. The cells were harvested and subjected to (A) western blotting for analysis of expression levels of the indicated proteins. Actin levels show equal total protein loading. (B) The cells kept for the indicated number of days in the DM were double stained with MitoTracker and Hoechst. Expression of MyoG or MyHC was visualized by immune-fluorescence. Representative micrographs shown were recorded by both bright field phase-contrast and fluorescence microscopy. MitoTracker, Hoechst, and MyoG or MyHC signals are shown in red, blue, and green respectively (scale bar in = 50 µm).

**Figure 4:**
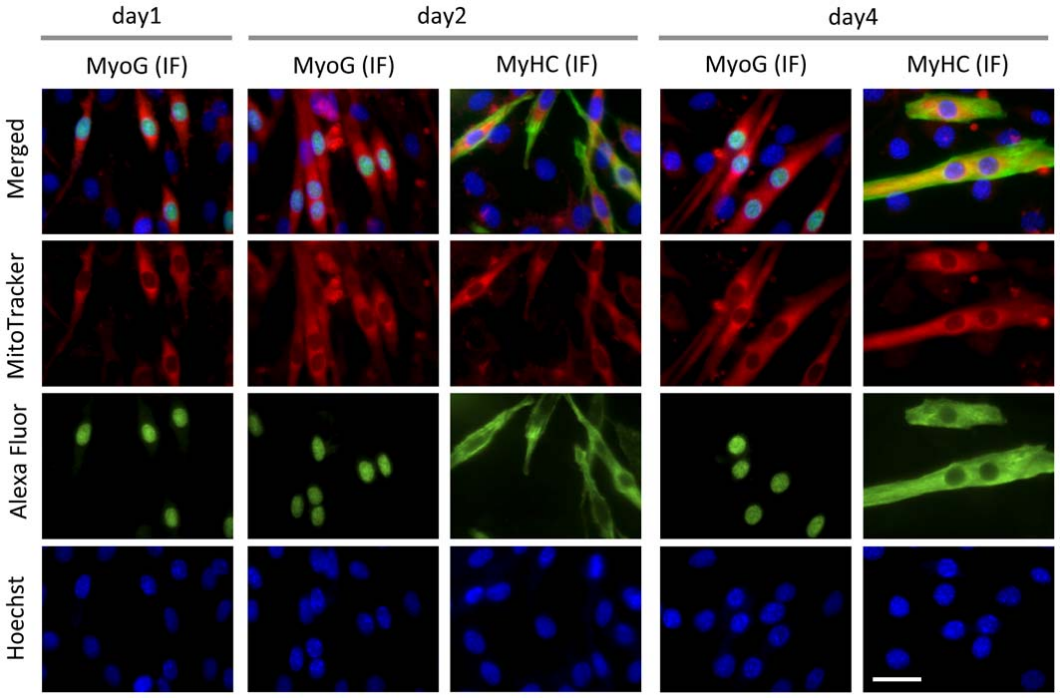
MyHC and MyoG positive cells are also MitoTracker positive. C2C12 cells were seeded on glass-bottom dishes. Upon reaching confluence, the cells were kept in DM for the indicated number of days to induce MT formation. Cells were subsequently double stained with MitoTracker and Hoechst as described above. Expression of MyoG or MyHC was visualized by immunofluorescence. Representative micrographs shown were recorded by fluorescence microscopy. MitoTracker, Hoechst, and MyoG or MyHC signals are shown in red, blue, and green respectively (scale bar in = 10 µm).

To confirm that MTP cells were differentiating cells, we employed a biochemical approach to identify expression of muscle specific marker proteins. Figure 3A shows a typical expression pattern of muscle specific proteins by immuno-blotting analysis and corresponding morphological changes. A well established differentiation marker, MyoG, was expressed at day1after induction of differentiation by DM and reached very high levels at day2 even though most of the cells were still mono-nucleated at this point. MyHC accumulation started at day2 when the cells began fusing and forming multi-nucleated MTs. Therefore, MyoG and MyHC serve as useful early and late markers of differentiation, respectively. To compare MPTs with MyoG or MyHC positive cells, a triple staining approach was used. First, mitochondria and nuclei were stained with MitoTracker and Hoechst 33342 in living culture conditions, and then the cells were fixed at day4 and analysed for MyoG or MyHC expression by immune-fluorescences (IF). MyoG nuclear expression (green) was detected in the cells showing elongated mono- and multi-nucleated cells, and MTPs were almost completely matched with MyoG positive cells (representative micrographs are shown in Figure 3B). In addition, all MyHC positive cells were MTP cells, but we occasionally found that MTP cells did not express detectable amount of MyHC by IF (Figure 3B). Since changes in the MitoTracker reactivity in differentiating cells become distinguishable as MyoG was up-regulated, up-regulation of mitochondrial biogenesis may occur earlier than MyHC accumulation and concomitant with MyoG induction. To analyse this in detail, we fixed cells at day1, 2, and 4 and performed triple–staining (MyoG, mitochondria, and MyHC). The higher magnification micrographs clearly indicate MyoG, MyHC, or MitoTracker positive cells (Figure 4), and almost all MyoG positive (>95%) and MyHC positive (>98%) cells were also MTPs throughout the course of the differentiation (Figure 4 and Table 1). Therefore, although small numbers of the MTPs were neither MyoG nor MyHC positives, MitoTracker can be used to detect majority of differentiating cells in the early and late stages of differentiation.

**Table 1:** Quantification of correlation between MyoG or MyHC positive cells and MitoTracker positive cells.

(% of nuclei)	day1	day2		day4	
	MyoG	MyoG	MyHC	MyoG	MyHC
MyoG&MitoTracker double positive/MyoG positive	95.5	100.0	N/A	96.5	N/A
MyoG&MitoTracker double positive/MitoTracker positive	85.1	84.2	N/A	84.6	N/A
MyHC&MitoTracker double positive/MyHC positive	N/A	N/A	98.1	N/A	100.0
MyHC&MitoTracker double positive/MitoTracker positive	N/A	N/A	86.7	N/A	80.2

Myotubes during differentiation were stained with MitoTracker and Hoechst and MyoG or MyHC. The total numbers of nuclei in MyoG-positive, MyHC-positive, and MitoTracker-positive cells were counted using each fluorescence micrographs (n = 12). The total numbers of nuclei in double (“MyoG and MitoTracker” or “MyHC and MitoTracker”) positive cells were also counted using merged micrographs (n = 12). The percentage of total number of nuclei in each double positive cell over the total number of the nuclei in indicated single positive cells was calculated.

### MyoD-converted myocytes from fibroblasts can be identified by MitoTracker

**Figure 5:**
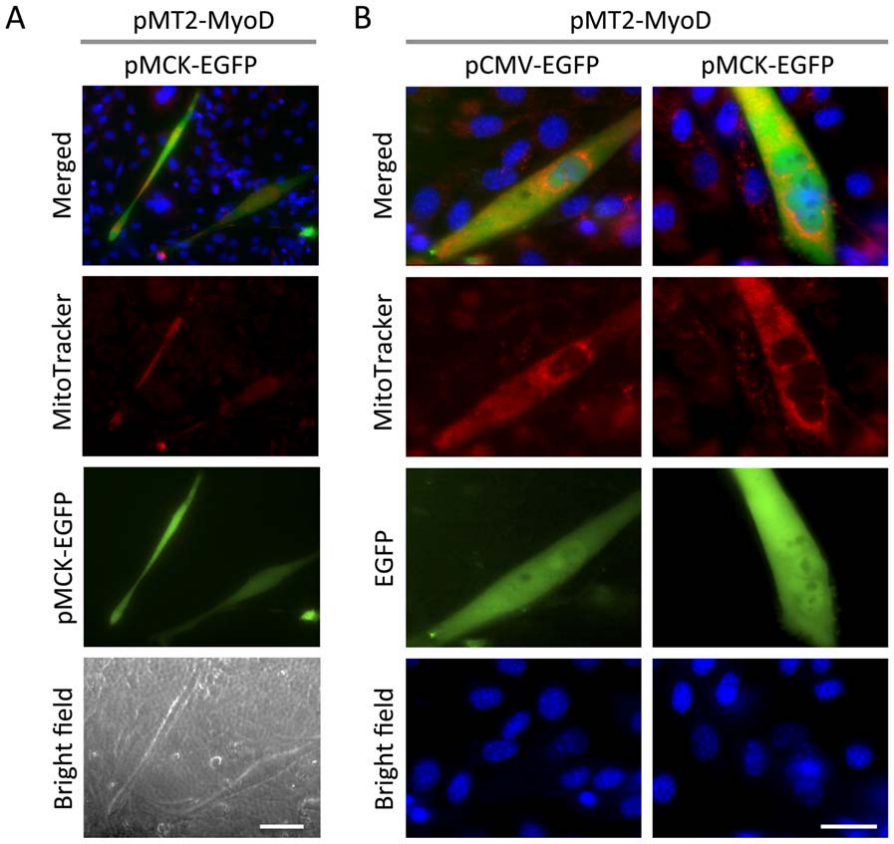
MyoD-converted myocytes from fibroblasts can be identified by MitoTracker. MyoD expression vector (pMT2-MyoD) or empty vector (pMT2) with either pCMV-EGFP or pMCK-EGFP was co-transfected into C3H10T1/2 fibroblasts. The cells were allowed an initial 2 days recovery in GM, followed by DM for 2 days. Nuclei and mitochondria were live-stained with Hoechst and MitoTracker and EGFP signal was recorded by fluorescence microscopy (scale bar in A = 50 µm, B = 10 µm).

Since MyoD expression is sufficient to convert fibroblast into myocytes [8], the possibility of whether these reprogrammed myocytes could be identified by MitoTracker staining was tested. C3H10T1/2 fibroblast cells were forced to differentiate into MTs by ectopic expression of MyoD. Transfected cells were marked and differentiation was monitored by EGFP reporter expression driven by muscle specific creatin kinase (MCK) promoter (pMCK-EGFP). After 2days in DM, MyoD expressing cells were elongated and multi-nucleated and became MCK positive (Figure 5A). MitoTracker reactivity was distinguishably higher in the cells showing morphological and biochemical changes. In a detailed analysis, non-transfected cell (EGFP negative cells) did not show any morphological changes typical of myocytes. More importantly, there was no MitoTracker reactivity observed in the absence of MyoD-conversion (Figure 5B). On the other hand, MyoD expressing C3H10T1/2 cells marked either by pCMV-EGFP or pMCK-EGFP, showed multi-nucleated MTs, induced MCK promoter activity, and became clearly visible MTPs. Therefore, these results demonstrate that MitoTracker staining is a highly efficient method to detect differentiating myocytes.

### Mitochondrial activity responds to a positive or a negative regulator of myogenesis

**Figure 6:**
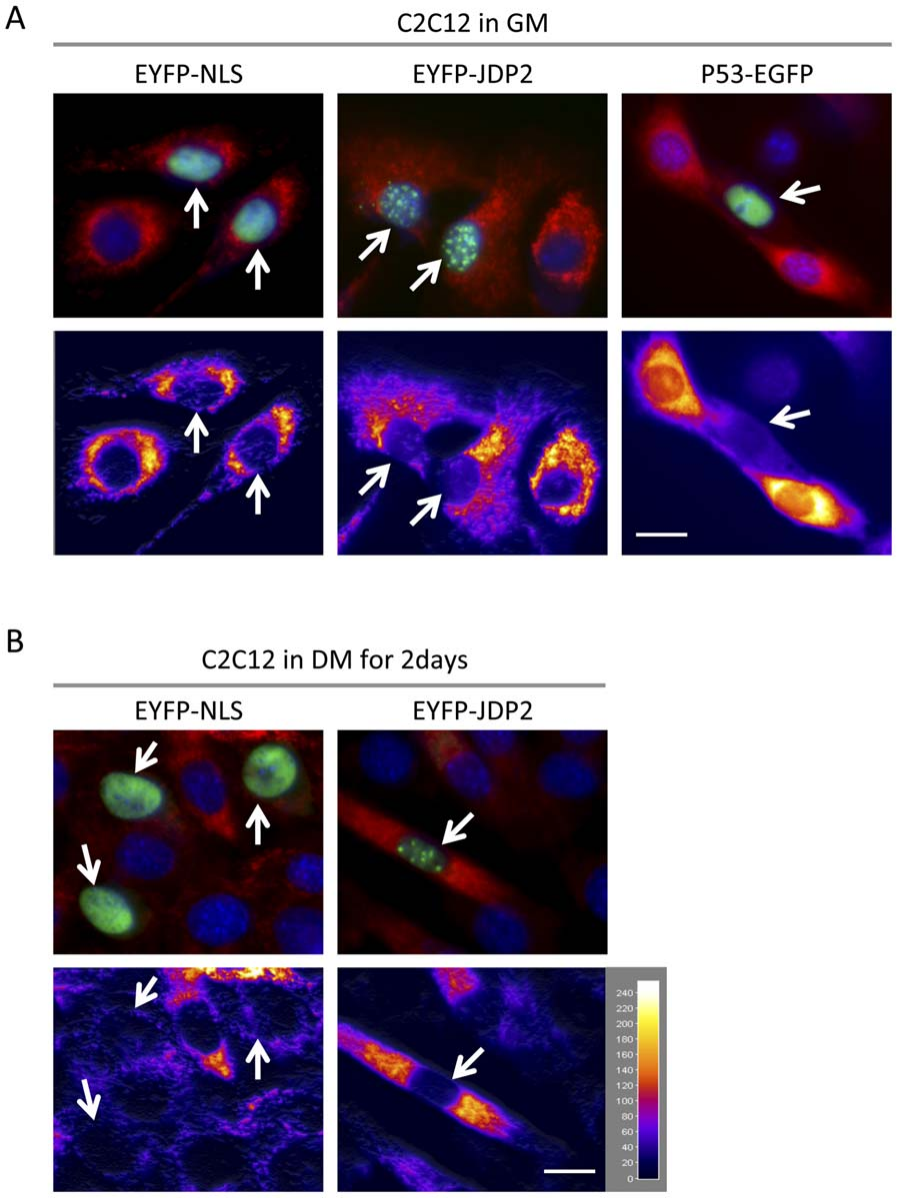
Mitochondrial activity responds to a positive or a negative regulator of myogenesis. C2C12 cells were transfected with p53-EGFP, EYFP-JDP2, or EYFP-NLS. Expression of fusion proteins, mitochondria (MitoTracker), and nuclei (Hoechst 33342) were visualized by fluorescence microscopy as described in Methods section. Micrographs were taken at 24 hrs after transfection (cells were in GM for 24 hars) in A, or 72 hrs after transfection (cells were kept in 48 hrs in DM) in B. MitoTracker, Hoechst, and transfected fusion proteins signals are shown in red, blue, and green respectively. White arrow indicates cells expressing an indicated fluorescence fusion protein. Merged image is shown in the middle panel. Intensity of MitoTracker signals were measured using ImageJ program (NIH) (top panel) based on the MitoTracker signal (Bottom panel). Micrographs shown are representative of the samples (n≥6) (scale bar = 10 µm).

In order to confirm the positive correlation between mitochondrial activity and muscle differentiation, known negative and positive regulator proteins for myogenesis were expressed ectopically, and their effect on mitochondrial reactivity for MitoTracker was measured. To minimize the “cross-talk” between green or yellow fluorescence to red fluorescence signal from cytoplasm (mitochondria), we choose nuclear localized p53 and JDP2. P53 is up-regulated in both aged muscle and muscle atrophy [9], [10], and required for inhibition of myogenesis by TNFα [11], [12]. In addition, p53 was identified recently as a direct negative regulator for mitochondrial biogenesis by inhibiting a master regulator, PGC1α [13]. JDP2 was originally identified as a c-Jun interacting nuclear protein [14] and later characterized as an enhancer for skeletal myogenesis [15]. To measure mitochondrial reactivity with MitoTracker staining in living cells, we transfected p53-GFP, EYFP-JDP2, or EYFP fused with SV40 nuclear localization signal (EYFP-NLS) construct [16] to proliferating MBs to mark transfected cells and stain mitochondria by MitoTracker. As seen in Figure 4, most of the p53-GFP, EYFP-JDP2, and EYFP-NLS fusion proteins were localized in the nucleus in transfected C2C12 cells as reported previously [12], [14], [16], [17]. MitoTracker staining revealed that EYFP-NLS or EYFP-JDP2 expression had no apparent effect on mitochondria activity in C2C12 in GM (Figure 6). However, p53-EGFP expressing cells had reduced mitochondrial reactivity compared to the untransfected (GFP-negative) cells. It was noted that much longer exposure was required to detect fluorescent signals from mitochondria in the MBs. After 2 days in DM, C2C12 cells started elongating and forming multi-nucleated myotubes, and as seen above, the cells indicating early differentiation phenotypes exhibited relatively higher mitochondrial activity (Figure 6B). In this condition, EYFP-JDP2 but not all EYFP-NLS expressing cells were elongated and had higher mitochondrial activity compared to untransfected cells. p53-EGFP expressing cells were not observed (data not shown). Therefore, p53 expressing MBs did not differentiate into MTs and greatly reduced mitochondrial reactivity to MitoTracker. In contrast, EYFP-JDP2 expressing cells were prematually went into myogenic program of differentiation and concomitantly showed higher mitochondrial activity. These changes are clearly detected in living differentiating cells without fixation by a mitochondrial fluorescence dye with fluorescent microscopy. Thus, we demonstrated here that this method for assessing myogenesis is reliable and effective.

## Discussion

Here, we report a simple, quick and effective method to identify differentiating muscle cells based on mitochondrial activity with a cell-permeable fluorescent dye, MitoTracker. Since this method is quick and robust and involves minimal manipulation, it is highly applicable for many downstream applications. Since a variety of cell permeable low-toxic fluorescence DNA dyes are commercially available, for example we also used Hoechst 33342 and SYBR-green (data not shown), mitochondrial reactivity to the could be quantified by a fluorescence detector easily and standardized to the relatively constant DNA fluorescence signal. Furthermore, since this double staining method of living cell does not require lengthy multi-step manipulations, it could easily be applied to a high throughput format. For example, screening of libraries, including chemicals, genomic etc, which affect muscle regeneration or maintenance using a C2C12 cell model could be envisioned. Such an approach could potentially be used to identify drugs which regulate muscle maturation and growth in a variety of pathological and physiological contexts [18], [19], [20], [21].

In summary we report a highly robust and rapid method to identify differentiating skeletal muscle cells using a mitochondrial specific fluorescence dye. Since live cells can be visualized and would be sorted by differences in the fluorescence signal, this method provides a substantial advantage compared to other methods that require cessation of the culture and further manipulation.

## Materials and Methods

### Cell culture

C2C12 myoblasts and C3H10T1/2 fibroblasts were obtained from American Type Culture Collection (CLR-1772 and CCL-226) and cultured in growth medium (GM) consisting of 10% fetal bovine serum (Sigma, F10511) in high-glucose Dulbecco's modified Eagle's medium (Gibco) supplemented with 1% penicillin-streptomycin (Invitrogen) and MEM non-essential amino acids (Gibco, 11140) at 37°C and 5% CO_2_. Myotube formation was induced by replacing GM with differentiation medium (DM), which consisted of 2% FBS in Dulbecco's modified Eagle's medium supplemented with 1% penicillin-streptomycin and MEM non-essential amino acids.

### Mitochondria and Nuclei double staining

MitoTracker®Red CMXRos (Invitrogen, M7512) and Hoechst 33342 (Sigma, B2261) were added into the culture media at final concentrations of 50 nM and 1 µM respectively. The cells were incubated under normal culture conditions for 30 min, and then visualized by fluorescence microscopy (Axiovert 200 M; Carl Zeiss).

### Red fluorescent signal quantification by a plate reader

The same number of cells were plated onto 94-well plate (Nalge Nunc International; 165305), and 2 days following seeding, the cells were transferred to DM to induce muscle differentiation. At the required time points, the cells were stained with MitoTracker and washed twice with 1XPBS to remove phenol-Red containing medium. Red fluorescent signals were measured by a plate reader (Perkin Elmer; EnVision 2104 multilabel Reader).

### Immuno-blotting analysis

Total cellular protein extracts were prepared in Nonidet P-40 lysis buffer containing 0.1% Nonidet P-40, 150 mm NaCl, 1 mM EDTA, 50 mM Tris-HCl pH 8.0, 1 mM sodium vanadate, 1 mM PMSF, supplemented with a protease inhibitor mixture (Sigma, P-8340) as described previously [22].

### Sarcomeric Myosin Heavy Chain and Myogenin detection by IF

MitoTracker and Hoechst stained cells were washed with Phosphate-buffered saline (PBS, pH 7.6) and fixed with 90% methanol at −20°C for 6 min. After fixation, the cells were incubated in 5% FBS in PBS for 30 min at 37°C for blocking and then incubated for 60 min at room temperature with MF-20 (primary antibody for MyHC) or F5D (for MyoG) (DSHB, University of Iowa) in PBS. After incubation, the cells were washed three times with PBS and incubated for 60 min at room temperature with Alexa Flour® 488 goat anti-mouse IgG secondary antibody (Invitrogen, A11029). The cells were again washed three times with PBS. Images were recorded with a microscope (Axiovert 200 M; Carl Zeiss) with Quorum Angstrom Optigrid system (Quorum).

### Plasmids and transfection

The reporter construct pMCK-EGFP was a gift from A. Ferrer-Martinez (Universitat de Barcelona, Spain). pMT2-MyoD was provided by S.Tapscott [23]. P53-EGFP mammalian expression construct was obtained from Addgene (plasmid #11770) and described elsewhere [16]. EYFP-NLS was constructed of insertions of an ORF of the PCR amplified EYFP from pEYFP-N1 (Clontech) at Xho I/EcoR I site and a nuclear localization signal (NLS) sequence of the NLS of the large tumour antigen (PKKKRKVED; BK polyomavirus) at EcoR I/Hind III sites of pcDNA3.1(−) (Invitrogen) in the same reading frame. EYFP-JDP2 was constructed of insertions of an open reading frame of the PCR amplified EYFP from pEYFP-N1 (Clontech) at Hind III/EcoR I site and JDP2 ORF RT-PCR amplified cDNA from total C2C12 RNA extracted by TRIzol (Invitrogen) according to the manufacture's protocol into pcDNA3 (Invitrogen). All transfections were performed with lipofectamine 2000 (Invitrogen) according to the manufacturer's protocol.
